# Effect of Polar Amino Acid Residue Substitution by Site-Directed Mutagenesis in the N-terminal Domain of *Pseudomonas* sp. Phytase on Enzyme Activity

**DOI:** 10.4014/jmb.2003.03020

**Published:** 2020-04-23

**Authors:** Ga Hye Lee†, Won Je Jang†, Soyeong Kim, Yoonha Kim, In-Soo Kong

**Affiliations:** Department of Biotechnology, Pukyong National University, Busan, 48513, Republic of Korea

**Keywords:** Beta-propeller phytase, alkaline phytase, site-directed mutagenesis, phytate

## Abstract

The N-terminal domain of the *Pseudomonas* sp. FB15 phytase increases low-temperature activity and catalytic efficiency. In this study, the 3D structure of the N-terminal domain was predicted and substitutions for the amino acid residues of the region assumed to be the active site were made. The activity of mutants, in which alanine (A) was substituted for the original residue, was investigated at various temperatures and pH values. Significant differences in enzymatic activity were observed only in mutant E263A, suggesting that the amino acid residue at position 263 of the N-terminal domain is important in enzyme activity.

Phytic acid is the main storage form of inositol and phosphorus in many plant tissues. It is a polyanionic molecule with six phosphate groups that combines with important minerals such as cobalt, copper, iron, manganese, zinc, and calcium to form insoluble phytate salts [1, 2]. Phytate salts are not available in monogastric (*i.e*., single stomach) animals, such as humans, pigs, poultry, and fish [3]. These animals lack intestinal phytases required for phytate hydrolysis during digestion, resulting in reduced bioavailability of minerals. Phytase, known as myo-inositol-hexakisphosphate phosphohydrolase, hydrolyzes indigestible phytate to liberate inorganic phosphorus. This enzyme has been used as a feed additive to enhance the nutritional value of phytate-rich, plant- based foods.

Phytase is divided into four types according to their catalytic and structural characteristics: beta-propeller phytase (BPP), purple acid phytase, histidine acid phytase, and cysteine phytase [4]. Among them, BPP has high thermal stability, substrate specificity, and neutral pH, which makes it suitable for industrial use [5].

Unlike BPP produced by *Bacillus* species, phytase of *Pseudomonas* sp. FB15 (PSphy) has an additional N-terminal domain. Our previous studies have reported that PSphy maintains high activity even at low temperatures [6]. Removal of the N-terminal domain from PSphy decreased the catalytic efficiency and activity at low temperatures [6], whereas fusion of the N-terminal domain with other *Bacillus*-derived BPP increased the catalytic efficiency and enzyme activity at low temperatures [7]. These results suggest the importance of the N- terminal domain of PSphy in low-temperature activity and catalytic efficiency.

This study was performed to identify amino acid residues that affect enzyme activity in the N-terminal domain of PSphy. Four amino acid residues (E140, Q172, E217, and E263), found in the region predicted as the active site, were chosen for testing. Selected amino acid residues were substituted using site-directed mutations and investigated for altered enzyme activity.

In general, the active site of BPP is at the propeller top (PT) region [6]. The 3D structure of PSphy was predicted using SWISS-MODEL (https://swissmodel.expasy.org/) to select amino acid residues in the PT region of the N- terminal domain as substitution residues. The PT region of the N-terminal domain consists of beta sheets, where the polar residue E140, 217, 263 and the hydrophilic residue Q172 are located (Fig. 1A). The amino acid residues were changed to alanine (A), a hydrophobic residue, to predict structural changes (Fig. 1B), and further studies on enzyme activity were conducted.

*Escherichia coli* DH5α and BL21(DE3) were used as hosts for gene cloning and protein overexpression. Recombinant *E. coli* was incubated at 37°C and overexpressed at 25°C in Luria broth medium supplemented with ampicillin at 100 μg/ml. pET-22b(+) was used as the vector for cloning.

Site-directed mutagenesis was performed using the overlap extension method as described in a previous study [7]. Briefly, forward and reverse fragments containing the mutation site were amplified by PCR. Additional PCR was conducted using the mixture of forward and reverse fragments as templates. The overlap extension PCR products were cut with restriction enzyme and then ligated with pET-22b(+) vector. *E. coli* DH5α was transformed and cultured in Luria broth agar medium containing ampicillin. Gene mutation in transformed colonies was confirmed through DNA sequencing.

Overexpression and purification of recombinant protein was performed as described in a previous study [6, 7]. The purified enzyme was analyzed by sodium dodecyl sulfate-polyacrylamide gel electrophoresis and stained with Coomassie Blue. Molecular weights were observed near 70 kDa (Fig. 2).

PSphy activity was measured by the molybdate-blue method with some modification [8]. Next, 50 μl of enzyme solution was mixed with 200 μl of substrate solution (1 mM Phytic acid sodium salt hydrate, Sigma-Aldrich, USA) and incubated at 40°C for 30 min. The reaction was terminated by adding 250 μl of 5% (w/v) trichloroacetic acid. The concentration of released inorganic phosphate was determined by adding 250 μl of coloring reagent (1.2% ammonium molybdate, 0.54% ferrous sulfate and 3.5% sulfuric acid). Absorbance was measured at 700 nm (Optizen POP, Korea).

The relative activity of PSphy was measured at various temperatures and pH values. In all experiments, the substrate concentration was 1 mM and CaCl2 concentration was 4 mM.

Significant differences in enzyme activity at various temperatures (25–50°C) were observed only at E263A, in which alanine was substituted for the original residue at position 263 (Fig. 3). At the optimum temperature of 40°C, enzyme activity increased by 25.89%. The largest change occurred at 45°C, at which enzyme activity increased by 39.22%. Similarly, significant differences in enzyme activity at various pH values (3–7) were observed only at E263A. At the optimum pH 6, enzyme activity increased by 29.58%. The largest change occurred at pH 7, at which enzyme activity increased by 36.84%.

Until now, little research has been carried out to determine the properties of residues in the N-terminal domain of PSphy. Altering amino acid residues affects the optimum pH and activity of the enzyme [9]. The specific activity and stability of BPP are affected when the negative charge is reduced by replacing negatively charged residues distributed on the catalytic surface [10]. For PSphy, it may be more important to reduce the polar residue content of the N-terminal domain and increase the hydrophobic residue content in order to have a positive effect on activity. Increased hydrophobic interactions resulting from increased hydrophobic residues have been reported to increase enzyme stability and activity [11, 12]. For this reason, hydrophobic core packing is considered an important strategy for the industrial application of enzymes. Likewise, the prediction of intramolecular interactions of mutant E263A in this study resulted in the formation of new hydrophobic interactions and increased activity compared to wild type at various temperatures and pH values (Fig. 4).

In conclusion, this study investigated altered enzymatic activity by substituting polar amino acid residues based on the structural prediction of the PSphy N-terminal domain. Enzyme activity was increased by replacing amino acid residue E263 with alanine, indicating that position 263 of the N-terminal domain plays an important role in enzyme activity. Further studies are needed to explain additional functions, active sites, and mechanisms of action of the N-terminal domain.

## Figures and Tables

**Fig. 1:**
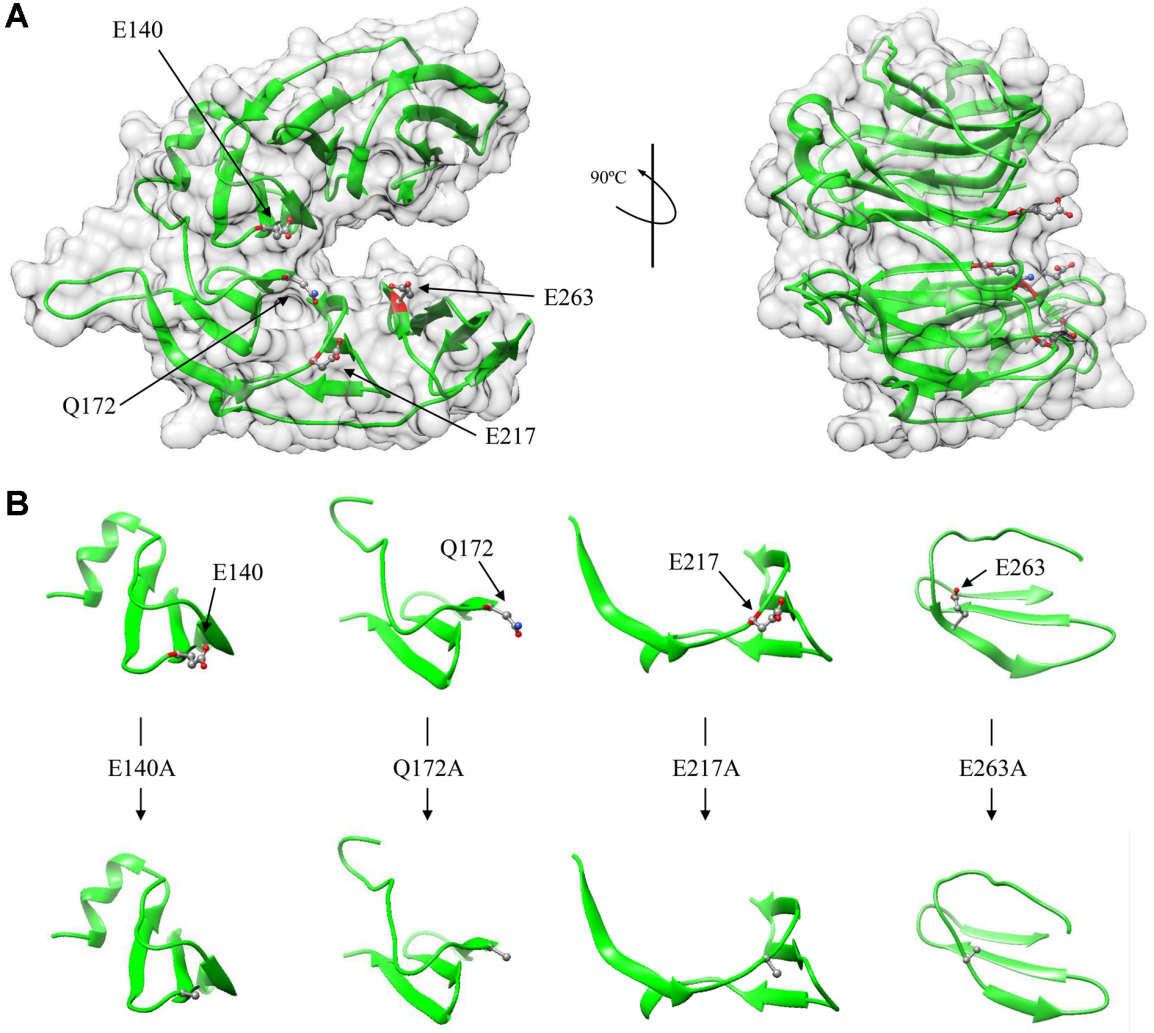
Prediction of 3D structure of (A) N-terminal domain and (B) amino acid substitution sites of *Pseudomonas* sp. FB15 phytase. The N-terminal domain is propeller shaped and 4 substitution residues are located in the top region of the propeller.

**Fig. 2:**
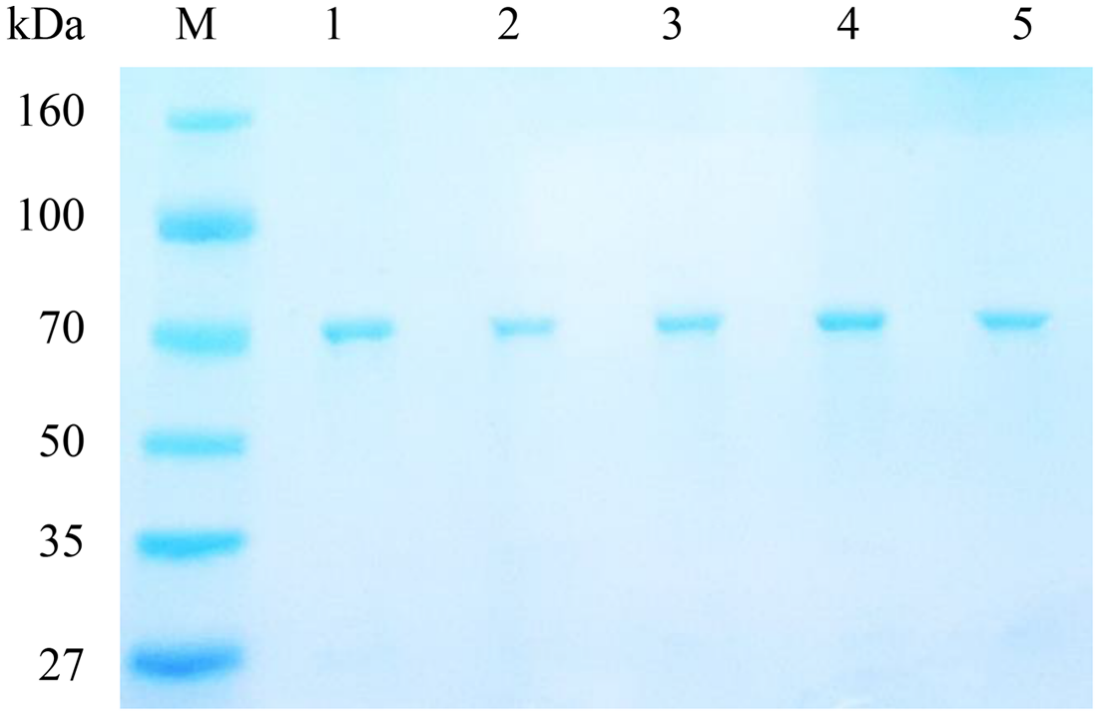
Sodium dodecyl sulfate-polyacrylamide gel electrophoresis analysis of the purified recombinant phytase. Lane M, molecular weight marker; Lane 1, wild-type; Lane 2, E140A; Lane 3, Q172A; Lane 4, E217A; and Lane 5, E263A.

**Fig. 3:**
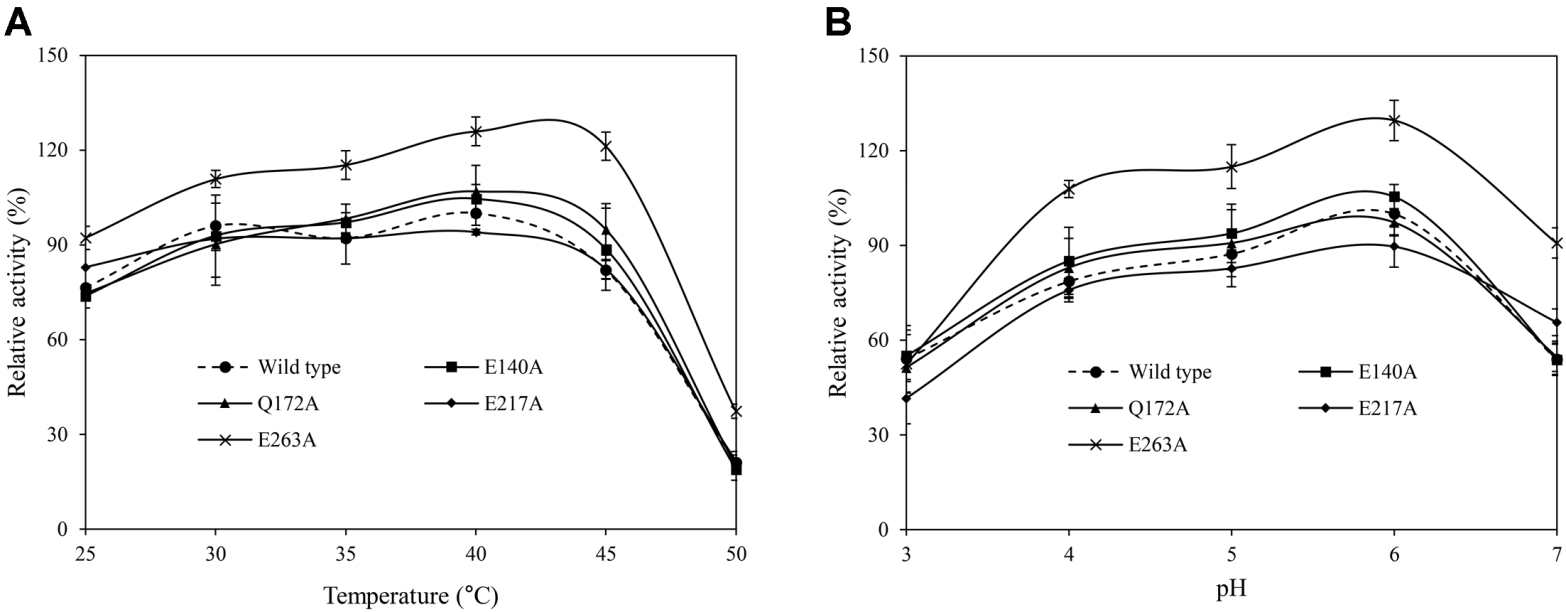
Effects of various (A) temperatures and (B) pH values on wild-type and mutant phytase activity. The activity was measured using 1 mM sodium phytate as a substrate in the presence of 4 mM CaCl2. Relative activity was expressed based on the activity of the wild-type under optimal conditions (40°C, pH 6). Significant differences (*p* < 0.05) in enzymatic activity were observed only in mutant E263A.

**Fig. 4:**
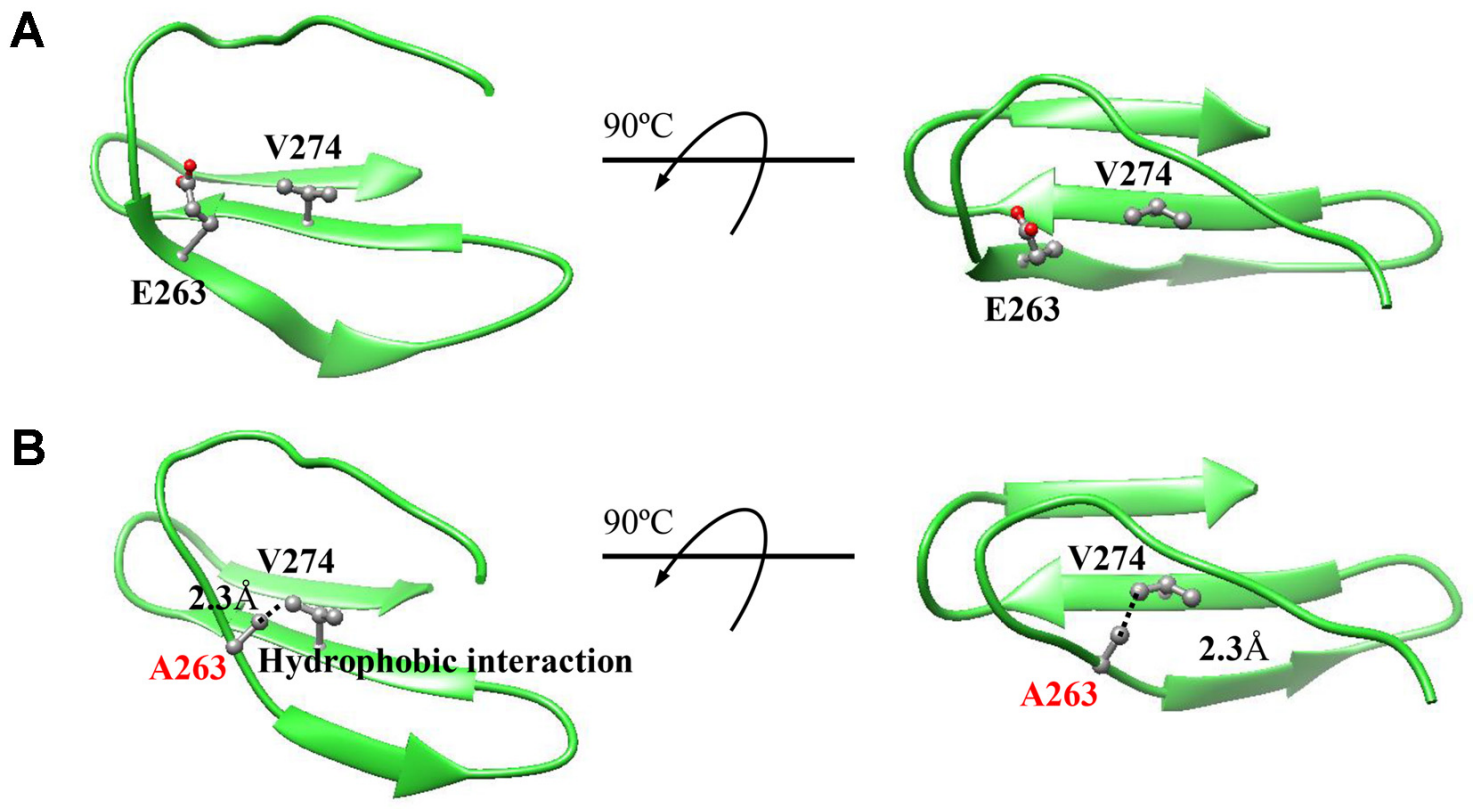
Schematic diagram of intramolecular interactions of (A) wild-type and (B) E263A. The prediction of intramolecular interactions of mutant E263A resulted in the formation of new hydrophobic interactions.
